# Temporal gene expression during asexual development of the apicomplexan *Sarcocystis neurona*

**DOI:** 10.1128/msphere.00111-24

**Published:** 2024-05-29

**Authors:** Sriveny Dangoudoubiyam, Jamie K. Norris, Sivaranjani Namasivayam, Rodrigo de Paula Baptista, Naila Cannes do Nascimento, Joseph Camp, Christopher L. Schardl, Jessica C. Kissinger, Daniel K. Howe

**Affiliations:** 1 Maxwell H. Gluck Equine Research Center, Department of Veterinary Science, University of Kentucky, Lexington, Kentucky, USA; 2 Center for Tropical and Emerging Global Diseases, University of Georgia, Athens, Georgia, USA; 3 Department of Genetics, University of Georgia, Athens, Georgia, USA; 4 Institute of Bioinformatics, University of Georgia, Athens, Georgia, USA; 5 Department of Comparative Pathobiology, College of Veterinary Medicine, Purdue University, West Lafayette, Indiana, USA; 6 Department of Plant Pathology, University of Kentucky, Lexington, Kentucky, USA; University at Buffalo, Buffalo, New York, USA

**Keywords:** transcriptome, merozoite, schizont, *Sarcocystis*, apicomplexan parasites

## Abstract

Asexual replication in the apicomplexan *Sarcocystis neurona* involves two main developmental stages: the motile extracellular merozoite and the sessile intracellular schizont. Merozoites invade host cells and transform into schizonts that undergo replication via endopolygeny to form multiple (64) daughter merozoites that are invasive to new host cells. Given that the capabilities of the merozoite vary significantly from the schizont, the patterns of transcript levels throughout the asexual lifecycle were determined and compared in this study. RNA-Seq data were generated from extracellular merozoites and four intracellular schizont development time points. Of the 6,938 genes annotated in the *S. neurona* genome, 6,784 were identified in the transcriptome. Of these, 4,111 genes exhibited significant differential expression between the merozoite and at least one schizont development time point. Transcript levels were significantly higher for 2,338 genes in the merozoite and 1,773 genes in the schizont stages. Included in this list were genes encoding the secretory pathogenesis determinants (SPDs), which encompass the surface antigen and SAG-related sequence (SAG/SRS) and the secretory organelle proteins of the invasive zoite stage (micronemes, rhoptries, and dense granules). As anticipated, many of the *S. neurona* SPD gene transcripts were abundant in merozoites. However, several SPD transcripts were elevated in intracellular schizonts, suggesting roles unrelated to host cell invasion and the initial establishment of the intracellular niche. The hypothetical genes that are potentially unique to the genus *Sarcocystis* are of particular interest. Their conserved expression patterns are instructive for future investigations into the possible functions of these putative *Sarcocystis*-unique genes.

**IMPORTANCE:**

The genus *Sarcocystis* is an expansive clade within the Apicomplexa, with the species *S. neurona* being an important cause of neurological disease in horses. Research to decipher the biology of *S. neurona* and its host-pathogen interactions can be enhanced by gene expression data. This study has identified conserved apicomplexan orthologs in *S. neurona*, putative *Sarcocystis*-unique genes, and gene transcripts abundant in the merozoite and schizont stages. Importantly, we have identified distinct clusters of genes with transcript levels peaking during different intracellular schizont development time points, reflecting active gene expression changes across endopolygeny. Each cluster also has subsets of transcripts with unknown functions, and investigation of these seemingly *Sarcocystis*-unique transcripts will provide insights into the interesting biology of this parasite genus.

## INTRODUCTION

*Sarcocystis neurona* is an obligate intracellular parasite in the phylum Apicomplexa and the primary etiological agent of equine protozoal myeloencephalitis, a debilitating neurological disease affecting horses in the Americas (1). *Sarcocystis neurona* is also recognized as a pathogen in marine mammals such as sea otters (2–4), Pacific harbor seals (5, 6), and California sea lions (7). Additionally, clinical infections with *S. neurona* have been reported from multiple other hosts, including domestic dogs, cats, and ferrets (reviewed in reference 1). The parasite has a complex life cycle that involves opossums as the definitive host (8) and various small mammals as intermediate hosts (9–12). Horses and sea mammals become infected by ingesting *S. neurona* sporocysts in feed and water contaminated with feces from infected opossums. In these hosts, *S. neurona* propagates by asexual replication that involves switching between extracellular merozoites and intracellular schizonts, which are responsible for the pathology associated with myeloencephalitis. The asexual stages can be grown *in vitro*, providing easy access to parasites that can be used for experimental purposes to better understand the parasite’s biology.

**Fig 1:**
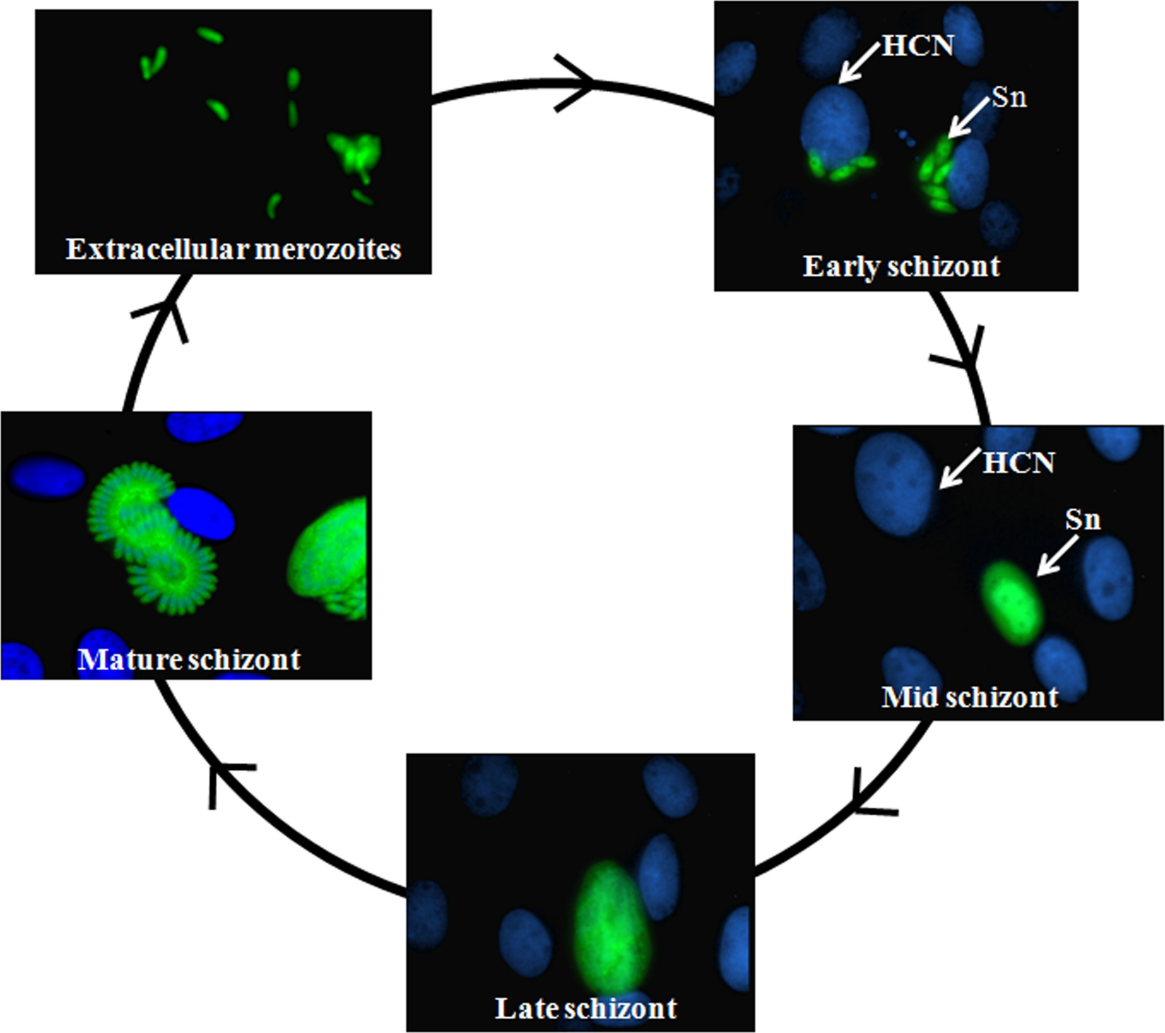
Progression of the schizont stages during the intracellular development of *Sarcocystis neurona* expressing yellow fluorescent protein. Post-invasion, the merozoites convert into schizonts that progressively develop into early-, mid-, and late-schizonts while undergoing a form of asexual reproduction called endopolygeny. In the final step in endopolygeny, the mature schizont forms 64 haploid merozoites fully equipped to egress and invade new host cells. *S. neurona*, Sn; DAPI-stained host cell nuclei, HCN.

Like the related parasites *Toxoplasma gondii* and *Neospora* spp., asexual propagation of *S. neurona* begins with invasion into a host cell. Once intracellular, however, *S. neurona* development differs appreciably from these evolutionary relatives. *Sarcocystis neurona* is found free in the host cell cytoplasm (13), in contrast with many coccidian parasites that reside within a parasitophorous vacuole (PV). Since the PV membrane serves as a protective interface between the developing parasite and the host cell (14, 15), the absence of a PV around *S. neurona* represents a fundamental biological difference in how this parasite interacts with the host cell to survive and replicate. In addition, intracellular development in *S. neurona* occurs via a complex process termed endopolygeny (13, 16, 17). This mode of cell division uncouples DNA replication from cell and nuclear division, resulting in a single polyploid nucleus inside a large mother cell (a.k.a. the schizont). The nuclear material in the mother cell eventually divides, concurrent with cytokinesis, resulting in the formation of multiple daughter merozoites (Fig. 1). Given the complexity and time required for endopolygeny (~72–84 h *in vitro*), the parasite must ensure coordinated regulation of gene expression to facilitate the accurate genesis, organization, and segregation of cellular components (e.g., organelles) into the nascent daughter zoites. Once these newly formed merozoites escape the host cell, their primary purpose is to find and invade new host cells. Based on the changing functional and metabolic needs across asexual development, it is anticipated that sets of genes in *S. neurona* are present at different levels between the extracellular, invasive merozoite stage and the intracellular, reproductive schizont stage of the parasite.

Publication of the *S. neurona* genome (18) established a foundation to accelerate gene discovery. Also, it facilitated comparative genomic analyses with other apicomplexans that reiterated the phylogenetic distinctness of the genus *Sarcocystis*. The genome sequence provided a wealth of information regarding gene content, including genes potentially unique to *S. neurona* or the genus *Sarcocystis*. However, little is known about gene expression during asexual development. To understand the changes in transcript levels during asexual development, we generated RNA-seq data from extracellular merozoites and intracellular schizonts at various time points during endopolygeny. As an adjunct, proteomics data obtained from merozoites were used to confirm the expression of merozoite-abundant transcripts. As documented previously for other apicomplexans (19–21), our analyses verified differential transcriptional regulation during asexual development, including genes encoding proteins predicted to be important for the invasive merozoites (e.g., secreted pathogenesis determinants or SPDs) and proteins necessary for intracellular growth and propagation (e.g., DNA replication, protein translation, etc.). Additionally, patterns of transcript regulation during parasite asexual growth are informative for transcripts encoding products of unknown function, including transcription of genes that are potentially unique to *S. neurona* or the genus *Sarcocystis*.

## MATERIALS AND METHODS

### *S. neurona* merozoite and schizont collection

*Sarcocystis neurona* strain SN3.E1 (ATCC PRA-420) was propagated in monolayers of bovine turbinate (BT) cells (22). Merozoites emerging from the lysed host cells were harvested by passing the culture lysate through 22-G and 25-G needles and filtering through a 3.0-µm Nucleopore membrane to collect merozoites free of host cell debris. The filtrate was centrifuged at 4°C to obtain merozoite pellets for the extraction of RNA. To obtain intracellular schizonts, BT monolayers in multiple flasks were infected with *S. neurona* merozoites at high multiplicity of infection to ensure that the parasites were greater than 50% of the culture biomass at the time of harvest. Samples from one tissue culture flask each were collected at 8, 24, and 48 h post-inoculation by treating the host cells with trypsin and centrifuging at 600 × *g* at 4°C to obtain infected cell pellets; these parasites were considered as early-stage (8 and 24 h) and mid-stage (48 h) schizonts. Samples from cultures at approximately 72 h post-inoculation, when a majority of late-stage schizonts were observed within the BT cell monolayer, were collected by scraping the BT cells from the flask and centrifuging at 4°C to obtain the infected cell pellet. All collected schizont samples were washed once with pre-chilled PBS and then used for total RNA extraction.

### RNA extraction and sequencing

Total RNA from duplicate samples of schizonts representing early-stage schizonts [8 h (UKY2a, UKY2b)], [24 h (UKY4a, UKY4b)], mid-stage schizonts [48 h (UKY5a, UKY5b)], late-stage schizonts [72 h (UKY6a, UKY6b)], and extracellular merozoites (UKY7a, UKY7b) was isolated using Trizol reagent (Life Technologies, Grand Island, NY, USA). Total RNA in nuclease-free water was submitted to the Genome Sequencing and Analysis Core Resource at Duke University (Durham, NC, USA) to generate strand-specific mRNA libraries. Bar-coded multiplexed libraries were sequenced on a single lane of the Illumina HiSeq 2000/2500 Rapid Run Platform to generate 100 bp paired-end reads.

### Bioinformatics protocols/analysis of the sequenced data

Steady-state transcript levels were analyzed between the two life cycle stages, merozoite and schizont (all time points). Additionally, temporal expression among the four schizont developmental time points (8, 24, 48, and 72 h) was analyzed to identify transcript abundance patterns during schizogony alone. Two different pipelines (Cuffdiff-TPM and DESeq2-normalized counts) were employed, and their output was compared. As a first pipeline, Trimmomatic v.0.33 (23) was used to remove any adapter or primer sequences. Bases with PHRED scores below 20 over a 5-bp sliding window were considered low quality and removed from the data set. Quality reports were generated for all the trimmed reads using FastQC v0.11.3. After filtering, only paired reads were retained for downstream analysis. Reads originating from BT cells (host cells used for parasite cultures) were removed by mapping to the *Bos taurus* reference genome and mitochondrial sequence, *UMD3.1* (GenBank accession: GCA_000003055.5) using the bwa tool v.0.7.12 (24). SAMtools v.1.3 and BEDtools v 2.24.0 were used in tandem with bwa to extract unmapped reads. These unmapped reads were presumed to be of *S. neurona* origin. TopHat v2.1.0 (25) was used to align reads from all stages to the SN3.E1 *S. neurona* reference genome (GCA_000727475.1). Cufflinks v2.2.1 was used to perform reference annotation-based transcript assembly with multi-read and bias correction. Cuffmerge was employed to merge all the individual assemblies, including any possible novel transcripts, with the original annotation. Cuffdiff was used to identify significant differences in the levels of the assembled transcripts between time points. The normalized values were expressed in transcripts per kilobase million reads (TPM) and were converted from the fragments per kilobase million reads values output obtained from the Cuffdiff program. A log_2_-transformed average fold difference of TPM values indicated whether the transcript levels varied between any two time points. A second pipeline wherein Hisat2 (v.2.1.0) (26) was used to align trimmed RNA-seq data to version 40 of the SN3.E1 reference genome from ToxoDB. HTSeq 0.11.2 (27) was used along with version 40 of the SN3.E1 reference genome annotation to quantify reads mapping to annotated genes. Files containing the raw counts were imported into DESeq2 1.32.0 (28) under an RStudio Server 1.4.1717 environment running R 4.1.1 on the Debian GNU/Linux 11 operating system. The normalized values were expressed as the base mean of reads that aligned to the gene plus/minus a log_2_ fold difference. A log_2_ fold difference of read values between any two samples and adjusted *P*-value generated by DESeq2 indicated differences in the gene transcript abundance (adjusted *P*-value ≤ 0.05).

Data on transcript levels from gene families of interest were used to generate heatmaps using R Studio 1.1.456 (29) and R 3.5.2 (30). Data were imported using the rJava 1.0-1 Java interface (31), the XLSX 0.6.0 package for R (32), and manipulated using the Reshape 3.5.2 package for R (33). Heatmaps were plotted using the ggplot2 3.3.6 package of R (34).

### BLASTX searches and functional prediction

To establish putative identities and/or refine the existing functional annotations from the *S. neurona* SN3.E1 reference genome sequence [EuPathDB.org, version 13 April 2015 (35)], Local BLASTX (v 2.11.0) (36) similarity searches were performed against NCBI’s non-redundant protein database (RefSeq release 91) by utilizing the download feature of the Blast2GO software (V. 5.2.5, java version 1.8.0_152) (37). *Sarcocystis neurona* sequences that did not show a match to any of the GenBank sequences in the protein database were classified as putative *Sarcocystis*-unique sequences. Functional prediction of the proteins encoded by transcripts was performed using Blast2GO (v.5.2.5, java version 1.8.0_152) and the Kyoto Encyclopedia of Genes and Genomes database (release 94) (38). Blast2GO-based functional enrichment analysis was performed on genes exhibiting differential abundance in merozoite and schizont stages. A list of over- and under-represented functions was generated using a *P*-value of 0.003 as the cutoff value. InterProScan (release 78) (39, 40) was used to predict the presence of signal peptides and domain/motifs in sequences previously identified as *S. neurona* unique and putative hypothetical proteins. Using default settings, ORFfinder v0.4.3 (41) was used to search for predicted open reading frames (of ≥25 aa) in these sequences without matches to sequences in NCBI’s non-redundant protein database.

### Clustering of schizont-abundant gene transcripts

Clustering was performed on genes whose transcripts were identified as abundant in the schizont stages. The pattern of these gene transcript levels and the numbers of *S. neurona*-specific hypothetical proteins present in each cluster from schizont data sets were analyzed. For c-means (fuzzy) clustering, the fuzzification parameter used to assign a probability of each gene being placed into a cluster was estimated using the “mestimate” function provided by the Mfuzz R package v2.56.0 (42). This method attempts to minimize the Gaussian fuzzifier while preserving the meaningfulness of the clustering (43). To optimize the number of clusters, given the number of time points and transcript levels, we calculated and compared the means of the sum of squared distances between cluster centroids and the pattern of all differentially abundant transcripts, weighted by the membership value of that transcript to that cluster. We allowed for 2–20 clusters and fuzzification values between 1.1 and 3, producing 380 different clustering conditions. Transcripts were then clustered based on their log_2_ fold change between merozoite and each schizont time point. A user-friendly interactive web-based app has been provided at https://Sarcocystis.shinyapps.io/SN3_Gene_Expression_Clustering/ to permit clustering with adjustable parameters, including the ability to adjust the minimum cluster membership value for each transcript to be placed in one cluster over another.

### Generation of a merozoite proteome

*Sarcocystis neurona* cultures in BT cell monolayers were set up to harvest merozoites. Proteome data were generated from whole merozoites and the excretory-secretory antigen (ESA) components released by the merozoites. Proteins from the whole merozoite were extracted by resuspending 10^8^ *S. neurona* merozoites in 100 mM ammonium bicarbonate and using a barocycler (Pressure BioSciences, Inc., South Easton, Easton, MA, USA). A portion of this sample was treated to remove lipids to collect protein samples with and without the presence of lipids (44). To collect the ESA fraction of the merozoites, parasites were harvested, washed, and resuspended in a secretion medium (RPMI with 10 mM HEPES with or without 3% FBS) containing 1% ethanol to stimulate secretion from apical organelles (45). This supernatant was run on SDS-PAGE, and bands were excised and digested to recover peptides. Samples from whole merozoites and the ESA fraction were analyzed on a nano Eksigent 425 HPLC system coupled to the Triple TOF 5600 plus (Sciex, Framingham, MA, USA). Data were acquired by monitoring 50 precursor ions at 250 ms/scan. Mascot Daemon v.2.4.0 (Matrix Science) was used for database searches against proteins annotated in the SN3.E1 *S. neurona* genome (35).

## RESULTS AND DISCUSSION

To gain insights into temporal expression during the asexual development of *S. neurona*, we generated substantial RNA-Seq data by Illumina sequencing from extracellular, invasive merozoites, and intracellular, replicative schizonts at various time points during endopolygeny (SRA PRJNA1006358). Approximately 55 million *S*. *neurona*-specific, paired-end reads were obtained from each of the merozoite and late schizont (72 h time point) stages after removing reads originating from the bovine host cells and other potential contaminants (Table S1). As expected, fewer *S. neurona-*specific reads were obtained from the 8 h (~11 million reads), 24 h (~17 million reads), and 48 h (~23 million reads) time points. Despite a high MOI, the schizonts are only a portion of the biomass in the cultures, and it is not possible to efficiently separate the schizont-stage parasites from host cells, as was done with the extracellular merozoites. Therefore, approximately 50%–85% of the raw reads generated from the early and mid-stage schizont samples originated from the host cells. The merozoite and late schizont stage sequencing reads mapped to the *S. neurona* genome at a 90%–97% rate, and ≤0.5% of the reads showed multiple alignments. Read mapping rates for the early- and mid-schizont stages were slightly lower at 81%–87%, but the number of reads that mapped to multiple loci remained low at less than 0.5%. Of the 6,938 genes annotated in the SN3.E1 *S. neurona* genome, 6,784 annotated genes were represented in the transcriptome. Of these, 4,111 genes exhibited significant differences in transcript levels between the merozoite and at least one time point of schizont development.

Both expression analysis pipelines (Cuffdiff-TPM and DESeq2-normalized counts) exhibited good agreement in identifying genes with differentially abundant transcripts (8–24 h: 93%, 24–48 h: 88%, and 48–72 h: 96%). As the Cuffdiff-TPM pipeline attempts to assemble transcripts and does not rely on previous gene annotation, novel genes were included in the analysis. In contrast, the DESeq2-normalized count’ analysis relies on genome annotation, so novel genes were excluded from the findings. With the added benefit of assessing non-annotated genes, the results provided by the Cuffdiff-TPM pipeline are reported here.

**Fig 2:**
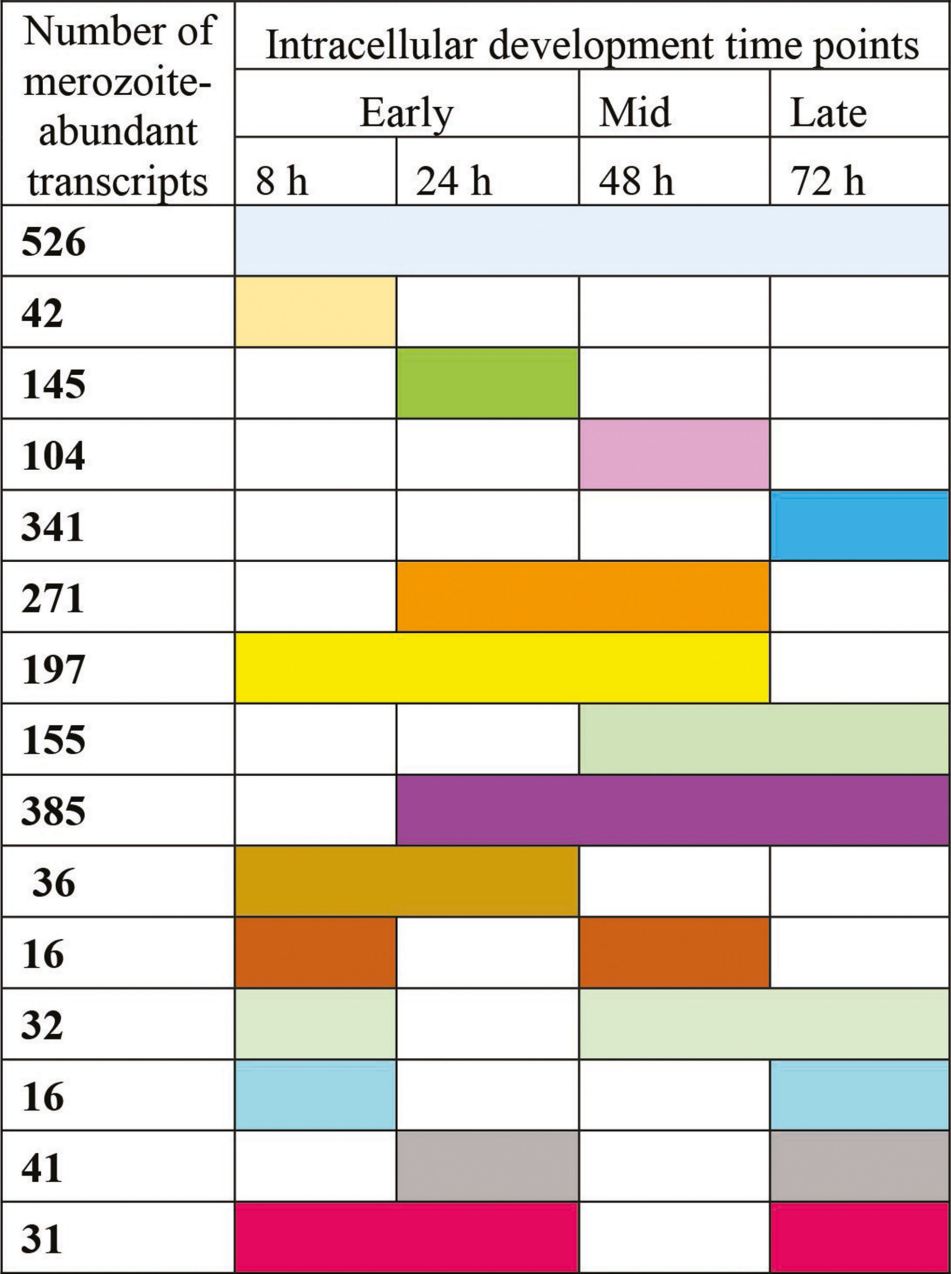
Heatmap showing the relative abundance of representative transcripts belonging to gene families important to the Apicomplexa. Average TPM values were calculated for each transcript across the different time points of schizont development, and the log_2_-transformed average fold differences as compared to merozoite (0 h) are presented for (A) secreted pathogenesis determinants, (B) cytoskeletal components, (C) AP2 transcription factors, (D) protein kinases, and (E) ubiquitin-proteasome pathway. The gene transcripts shown here are highlighted in the supplemental Table S4.

**Fig 3:**
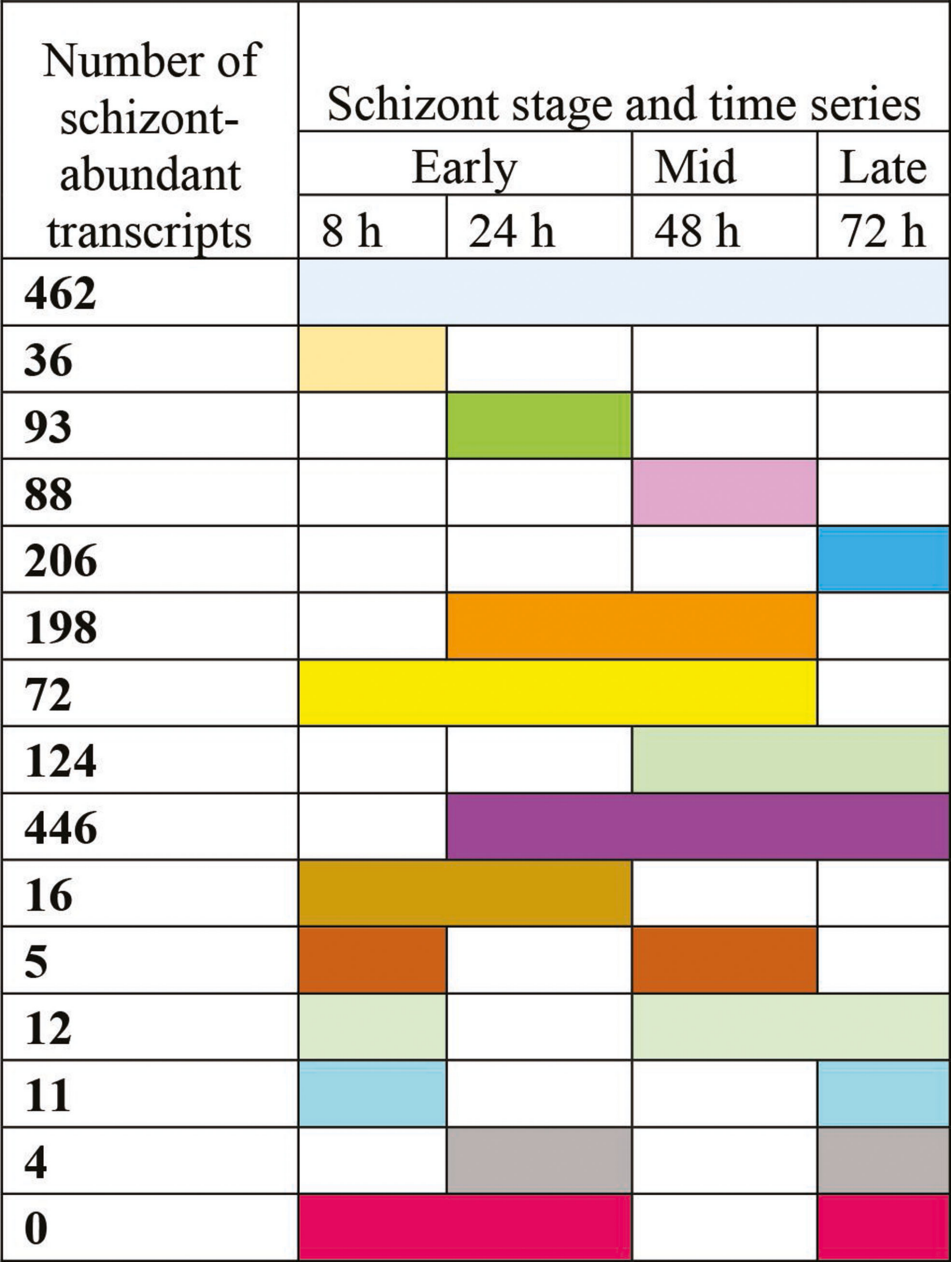
Differential abundance of transcripts in the schizont stage of *S. neurona*. The number of schizont transcripts found to be abundant when schizont transcriptome (individual or combination of time series) was compared against merozoite transcriptome is shown in each row. The different colors represent the time series combinations in which the schizont transcripts were abundant and correspond to the list of genes in each category found in Table S2 (also color-coded).

Analysis of steady-state transcript levels between merozoite and all schizont RNA-Seq data revealed significantly higher TPM values for 2,338 genes in the merozoite, while 1,773 genes showed significantly higher TPM in the schizont. The distribution of gene transcripts abundant in the merozoite relative to individual or different combinations of schizont time points is presented in Fig. 2. Likewise, the schizont gene transcripts that were significantly increased relative to merozoites are presented in Fig. 3. The list of genes, descriptions, and corresponding TPM values (base mean count and log_2_ fold difference values) of differentially abundant transcripts are provided in Table S2.

**Fig 4:**
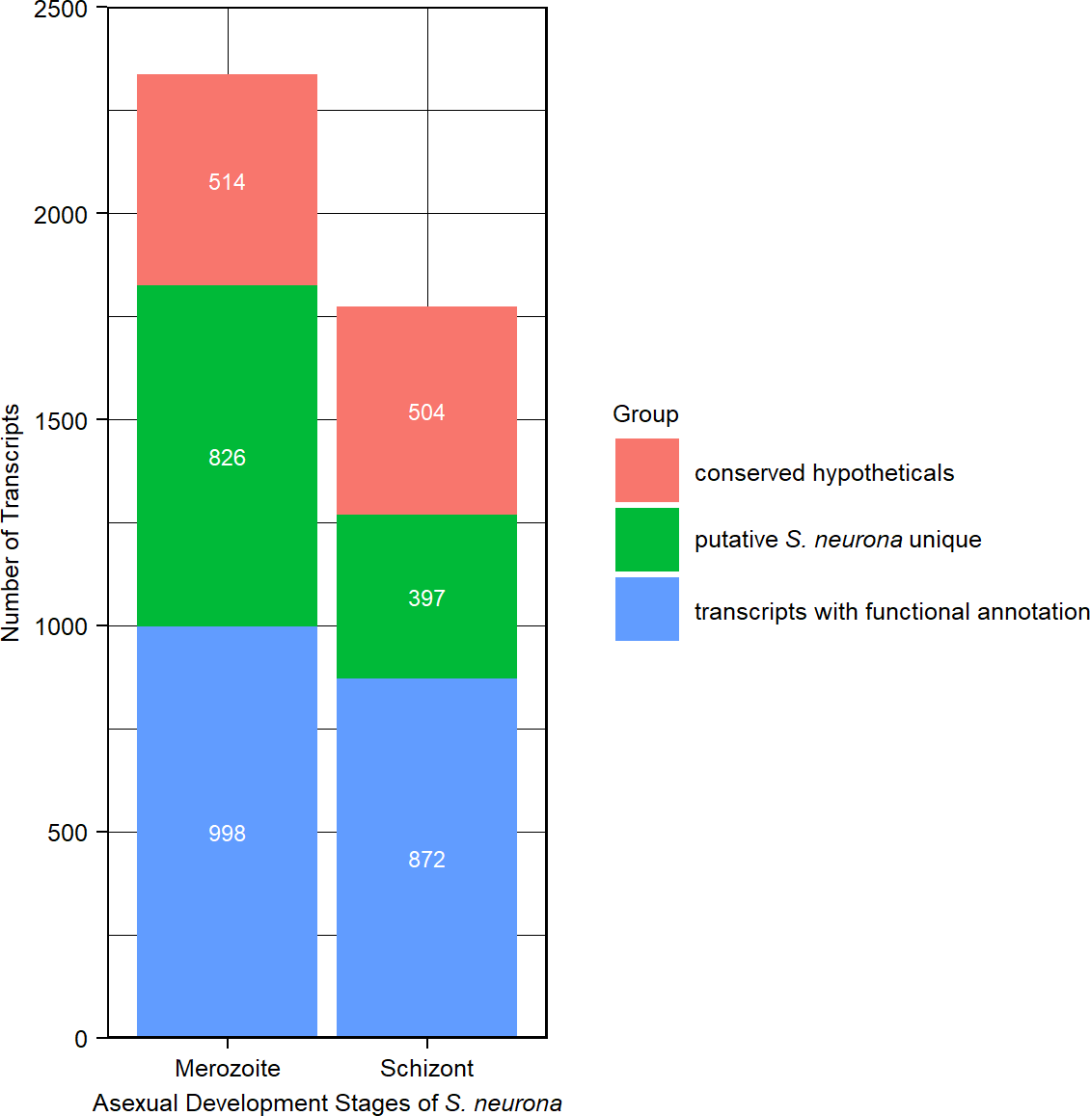
BLASTX analyses of merozoite and schizont genes. Searches against NCBI’s non-redundant protein database revealed putative *Sarcocystis*-unique sequences with no significant similarity to sequences in the public database.

BLASTX searches against NCBI’s non-redundant protein database provided a putative functional annotation for 998/2,338 merozoite-abundant transcripts and 872/1,773 schizont-abundant transcripts (Fig. 4). Similarity to conserved hypothetical proteins with no functional assignment from other organisms, including apicomplexans, was found for 514 and 504 of the merozoite- and schizont-abundant genes, respectively. Interestingly, the remaining 826 merozoite-abundant genes and 397 schizont-abundant genes had no BLASTX match to any sequences in the public databases, as was observed during the *S. neurona* genome analyses (18). These unique sequences represent putative *Sarcocystis*-specific genes that may be responsible for some distinctive characteristics (genome size, host range, number of species, replication method, intracellular niche, etc.) that define this parasite genus. The transcriptome information presented herein documenting temporal expression during *S. neurona* asexual development provides insights to investigate these *Sarcocystis*-specific genes further.

**Fig 5:**
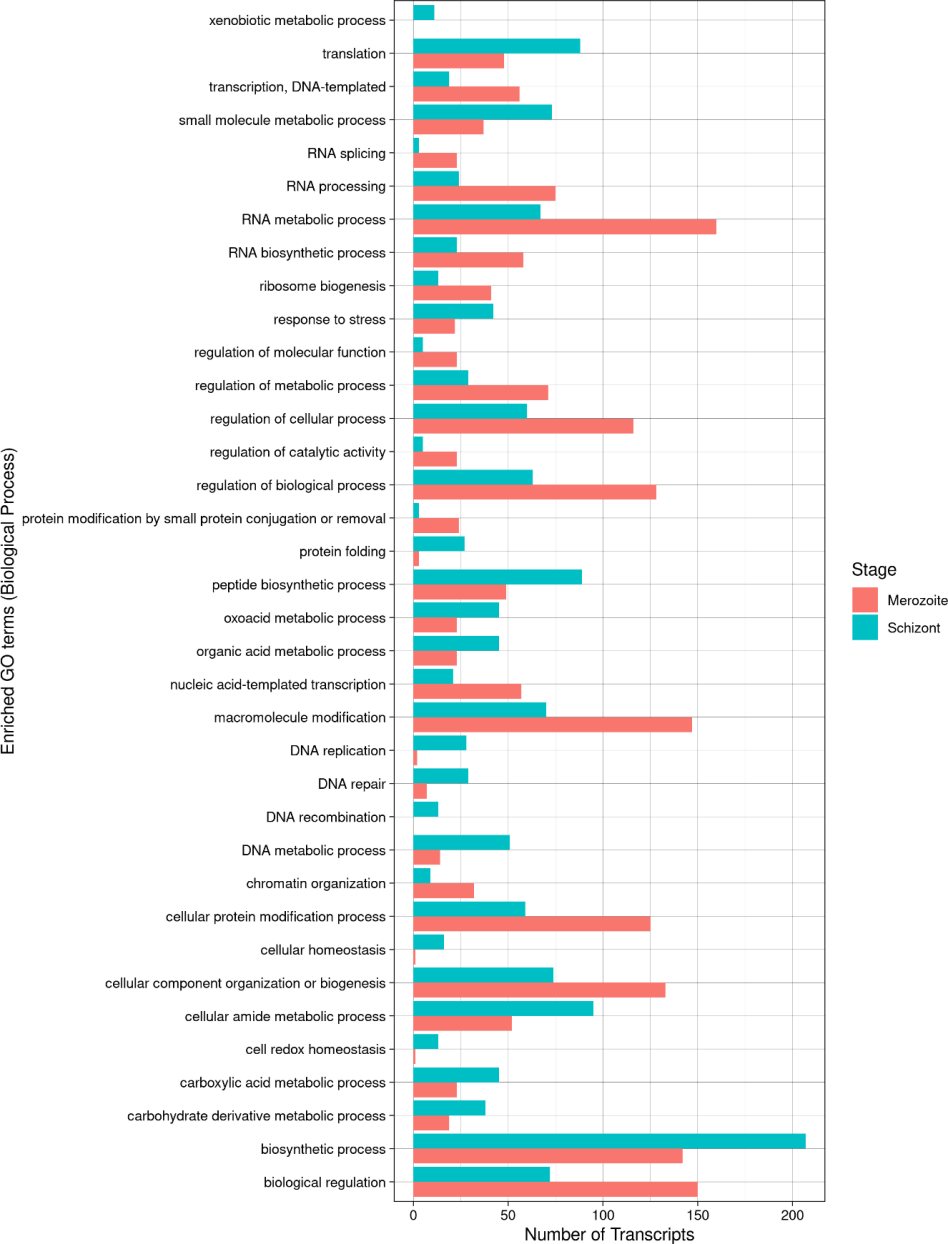
Gene ontology distribution between merozoite and schizont stages of *S. neurona* among genes exhibiting transcript level differences. GO terms related to biological processes (DNA, RNA, and energy metabolism, DNA repair and recombination) that show enrichment in either merozoite or schizont stages are presented.

Gene ontology (GO) assignments to the merozoite and schizont transcripts revealed distinct functional profiles between the two data sets (Fig. 5), as anticipated. In general, various energy metabolism and energy conversion processes were enriched in the schizont stage of *S. neurona*, while regulation of processes related to RNA metabolism and processing was enriched in merozoites. In the schizont stages, gene products associated with translation, protein folding, response to stress, cellular homeostasis, and various DNA processes such as DNA repair, recombination, and replication were enriched. These findings are consistent with intracellular schizonts undergoing endopolygeny, which would be expected to require all these processes. Interestingly, processes associated with chromatin structure and dynamics, transcription, RNA processing and modification, and ribosomal biogenesis were enriched in the extracellular merozoites. These results suggest that certain gene sets are transcribed in merozoites prior to host cell invasion to allow rapid protein production as the parasite transforms into the intracellular schizont stage and initiates endopolygeny.

As an adjunct to the transcriptome data, a partial proteome data set was generated from whole merozoites and the ESA fraction. These analyses revealed 1,061 *S*. *neurona* proteins from the whole merozoite extraction and 255 from the ESA fraction (Table S3). The proteome included multiple previously characterized proteins, such as surface antigen and SAG-related sequences (SAG/SRSs), surface protein 1 (SnSPR1), microneme (MIC) antigen 10 (SnMIC10), and apical protein 1 (SnAPR1). Comparing the transcriptome and proteome of the merozoite provided protein expression evidence (validation) for 348 genes highly expressed in the merozoite stage (Table S3). Among these, 67 were hypothetical proteins, of which 23 were identified in the merozoite transcriptome as putatively unique to *S. neurona*.

### Secreted pathogenesis determinants

**Fig 6:**
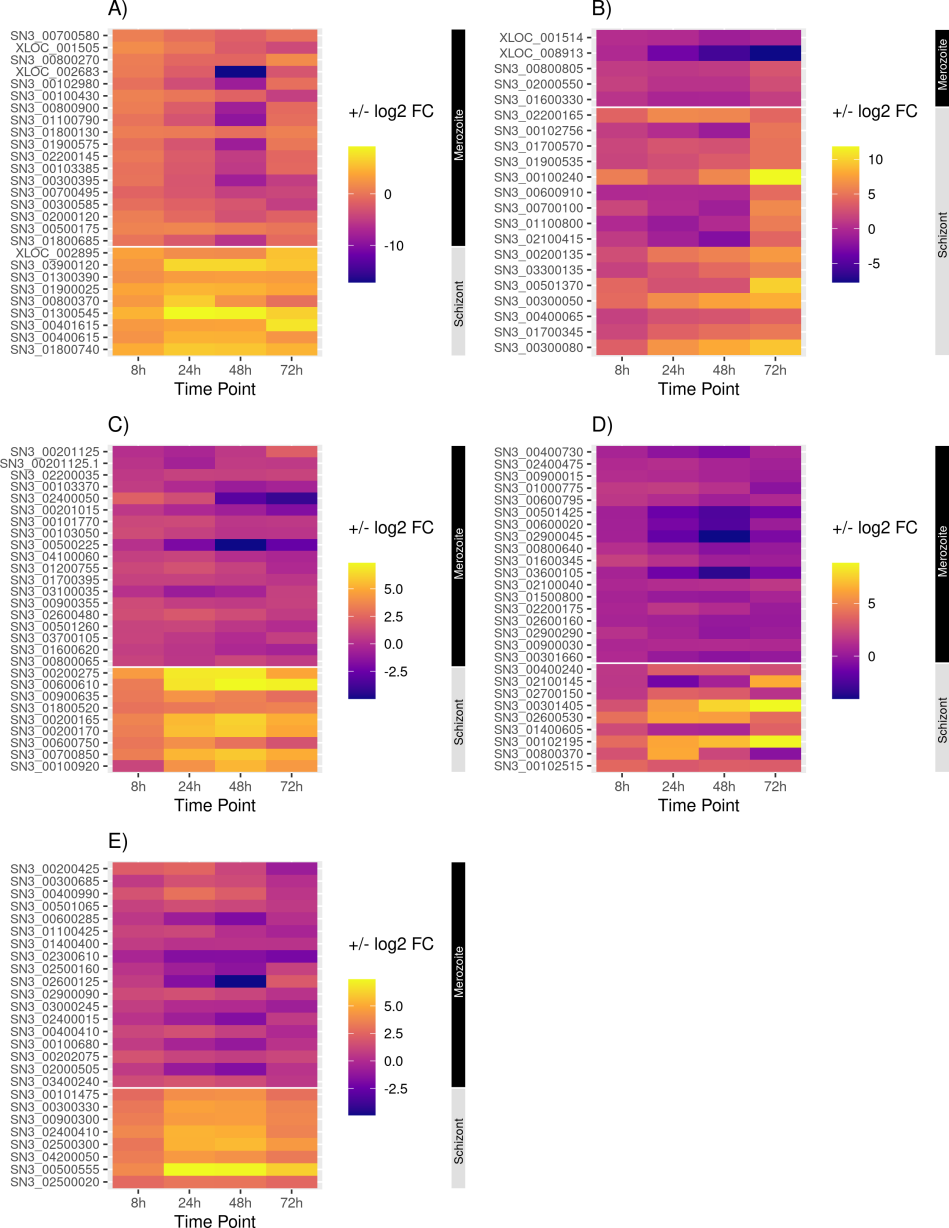
Heatmap showing the relative abundance of representative transcripts belonging to gene families important to the Apicomplexa. Average TPM values were calculated for each transcript across the different time points of schizont development, and the log_2_ -transformed average fold differences as compared to merozoite (0 h) are presented for (A) secreted pathogenesis determinants, (B) cytoskeletal components, (C) AP2 transcription factors, (D) protein kinases, and (E) ubiquitin-proteasome pathway. The gene transcripts shown here are highlighted in Table S4.

Due to their predicted contribution to parasite virulence, gene families encoding proteins termed secreted pathogenesis determinants (46) were examined for changes in steady-state mRNA levels across merozoite and schizont stages. These SPDs include the surface antigens (SAGs/SRS domain-containing proteins) and those secreted from the micronemes, rhoptries, and dense granules (Fig. 6A; Table S4). Although not absolute, SPDs are generally associated with functions needed during host cell invasion and the initial establishment of the intracellular niche. Consequently, it was predicted that many of the SPD transcripts would be more abundant in the merozoite transcriptome.

#### Surface antigens

The SAG/SRS gene family encodes glycosylphosphatidylinositol-anchored proteins that play a role in adhesion and invasion of the host cell and are likely involved in immune modulation or persistence within the host (47). Differential expression of SAGs across the sporozoite, bradyzoite, and tachyzoite/merozoite stages has been shown to occur in coccidian parasites, including *S. neurona* (48)*,* so we were interested in determining whether the SnSAG/SRS gene transcripts would exhibit differences during *S. neurona* asexual development. A difference in transcript levels was not observed for SnSAG1, SnSAG2, SnSAG3, and SnSAG4, consistent with detecting these four proteins throughout endopolygeny (49). Among the newly identified SRS proteins in *S. neurona*, transcripts for SRS53A/53C (SN3_00700580), 17 kDa surface antigen (SN3_00800270), and two SRS domain-containing proteins (non-annotated genes on scaffold00002:3686455–3688924 and scaffold00004:5469663–5474240) were significantly more abundant in the merozoites. The SnSRS53A/53C transcript was equally abundant in the merozoite and the early schizont (8 h) but significantly lower in the late-stage schizonts. Notably, transcripts for three SRS domain-containing proteins, SRS50 (non-annotated gene on scaffold00004:5469663–5474240), SRS 21 (SN3_03900120), and SRS domain-containing protein (SN3_01300390) were abundant in the schizont stages (Fig. 6A; Table S4). Although their TPM values were low overall, SRS50 (scaffold00004:5469663–5474240) and SRS21 (SN3_03900120) transcript levels were higher in at least one time point of the schizont than in the merozoite stage. Considering the potential involvement of SAG/SRS proteins in immune evasion (47), upregulation of the SnSAGs/SRS transcripts during the intracellular development could indicate a role in protecting the PV-lacking schizonts from innate immune responses and intracellular killing mechanisms in the host cell cytoplasm.

#### Microneme proteins

Micronemes are secretory organelles containing adhesive factors that facilitate attachment to and invasion of the host cells (50). An assortment of microneme protein transcripts [SnMIC14 (SN3_00102915), SnMIC12 (SN3_01600510), SnMIC10, MIC15 (SN3_00200915), three SnMIC8 paralogs (SN3_00100430, SN3_00400805, and SN3_02200145), SnMIC7 (SN3_02000450), three SnMIC4 paralogs SN3_01800610, SN3_01900575, and a non-annotated gene on Sn_small_contig04436:12–249, SnAMA1 (SN3_01000640), and several putative microneme proteins] were abundant in the merozoite stage (Fig. 6A; Table S4) consistent with the importance of these proteins for the invasion of host cells by zoites. Also, TPM values and normalized read counts of SnMIC10 (SN3_00102980) were found to be congruent with its experimentally established temporal protein expression during endopolygeny (51). Several of the MIC proteins [MIC10, two MIC8 paralogs, MIC13 (SN3_00800900), MIC4, and AMA1], as well as other microneme proteins designated as putative proteins, were evident in the merozoite proteome (Table S3). In *T. gondii,* the secretory serine protease subtilisin 1 (TgSUB1) is associated with micronemes and is involved in MIC protein processing (52). SnSUB1 (SN3_00101610) transcripts were abundant in the merozoite stage, and mRNA levels gradually decreased post-invasion until the late schizont stage (72 h time point), at which point the transcript levels increased. Subtilisin 11 (SN3_00100770), another serine protease that has not been functionally characterized*,* exhibited mRNA levels similar to SnSUB1, potentially reflecting similar functions (Fig. 6A; Table S4).

Rhomboids are intriguing apicomplexan membrane proteases with varied localizations and diverse biological functions. Transcripts encoding homologs of non-mitochondrial rhomboid proteins SnROM1 (SN3_00201570) and SnROM4 (SN3_03500070) were significantly more abundant in *S. neurona* merozoites and had the lowest transcript levels during mid-schizont stages (Fig. 6A; Table S4). In *T. gondii*, TgROM4 is involved in processing surface adhesins and facilitates invasion (53), while TgROM1 plays a role in intracellular development (54). Interestingly, significantly lower transcript levels of SnROM1 at the mid-schizont stage would suggest that this protein plays a biological role dissimilar to TgROM1. A SnROM6 (SN3_01900025) homolog was found to be differentially and increasingly abundant during schizont development. The biological role of TgROM6 in *T. gondii* is unclear (55).

#### Rhoptry proteins

Rhoptries are bulb-shaped organelles that secrete an array of proteins essential for invasion and host cell modification by apicomplexans (56). Despite the absence of identifiable rhoptries in *Sarcocystis* merozoites (57), transcripts for both rhoptry neck (RON) and rhoptry (ROP) proteins were found in the *S. neurona* transcriptome. In *T. gondii*, RON proteins are integral to the moving junction formed during parasite invasion (58, 59), and RON8 was found to facilitate the attachment of *T. gondii* to the host cell during invasion (60). Consistent with their expected role in host cell invasion, four RON homolog proteins, SnRON5 (SN3_00801030), SnRON6 (SN3_00600725), SnRON8 (SN3_02800355), and SnRON9 (SN3_04200095) showed significantly higher transcript levels in the merozoite stage. Transcripts of ROP protein homologs SnROP9 (SN3_00300585), SnROP14 (SN3_01800315), SnROP19 (SN3_01200585), SnROP20 (SN3_00202375), SnROP27 (SN3_02000120), SnROP24 (non-annotated gene scaffold00002:7511728–7515520), SnROP26 (SN3_00700495), SnROP39 (SN3_00300395), three SnROP40 (SN3_02000120, non-annotated genes scaffold00006:2870152–2873502 and scaffold00012:718179–719065) paralogs were abundant in the *S. neurona* merozoite. Transcripts for two additional rhoptry proteins, SnROP21 (SN3_00800370) and SnROP30 (SN3_01300545), also appeared to be temporally regulated (Fig. 6A; Table S4). Both TPM and normalized read counts suggested significantly higher transcripts of SnROP21 during the early schizont stage (24 h time point), while that of SnROP30 showed a significant increase in the mid-schizont (24 and 48 h) stages. SnRON3, SnROP9, SnROP21, SnROP26, and SnROP39 were found in the merozoite proteome (Table S3), providing evidence that these ROP proteins are expressed despite the absence of recognizable rhoptry organelles in *S. neurona* merozoites.

In *T. gondii,* subtilisin2 (TgSUB2) is involved in rhoptry protein processing (60). The *S. neurona* homolog SnSUB2 gene (SN3_00103385) was found to be differentially abundant with significantly higher transcript levels in the merozoite stage (Fig. 6A; Table S4). This protein was also identified in the *S. neurona* merozoite proteome (Table S3). Similar to TgSUB2, SnSUB2 may be involved in rhoptry protein processing. However, it needs to be determined whether SnSUB2 and SnROP proteins localize to the same compartment in *S. neurona* merozoites, given their lack of rhoptry organelles.

#### Dense granule proteins

Dense granules are secretory vesicles containing proteins traditionally thought to serve effector roles at the PV (61). Recent discoveries indicate that some dense granule (GRA) proteins are directed to the host cell nucleus, where they regulate gene expression (reviewed in reference 62), thus expanding the potential roles of GRA proteins. Fully mature *S. neurona* merozoites have 9–16 dense granules that appear to arise from the Golgi complex (13). In contrast to *T. gondii*, where the dense granule protein repertoire is extensive (>40 proteins), only a single *Sarcocystis* GRA protein has been described (DG32) (63), with just three additional GRA proteins (GRA9, GRA10, and GRA12) annotated in the SN3.E1 *S. neurona* genome. Of these four putative GRA proteins, GRA10 transcripts were significantly higher in the merozoite stage, while DG32 and GRA9 were schizont-abundant transcripts (Fig. 6A; Table S4). A *T. gondii* nucleoside triphosphate hydrolase, TgNTPase, is a dense granule protein (64) secreted into the parasitophorous vacuole during intracellular development of *T. gondii*. SnNTP1 exhibited a significant difference in transcript levels between merozoite and schizont stages, consistent with the previous findings that this protein may not serve a role during endopolygeny (65). While it is clear that *S. neurona* is rich in dense granule organelles, identifying so few GRA protein homologs suggests that the contents and collective functions of these secretory vesicles in this parasite could be distinct from its sister genera. The timing of GRA gene transcription combined with the lack of a PV during *S. neurona* intracellular development provides an interesting foundation for analyzing the composition and function of these proteins.

#### Toxolysins

Homologs of genes for the two *Toxoplasma* toxolysins, TgTLN4 (66) and TgTLN1 (67), were identified in *S. neurona* (Fig. 6A; Table S4). The transcript for SnTLN4 (SN3_01800685), a metalloproteinase of the micronemes, was abundantly present in the merozoite stage, as was the protein in the merozoite proteome analysis (Table S3). Transcript levels of this gene were much lower across all the schizont development time points but particularly low in early-mid schizonts (24 and 48 h time points). Interestingly, transcripts for a second toxolysin, SnTLN1 (SN3_01800740), were abundant in the schizont stages. Despite their low TPM and normalized read counts, SnTLN1 transcripts were 9–13-fold lower in the merozoite stage.

### Components of the parasite cytoskeleton

Irrespective of the mode of propagation (i.e., endodyogeny, endopolygeny, and schizogony) used by different species of apicomplexans, the formation of daughter cells within the mother cell is a complex and highly coordinated process (reviewed in reference 68). The inner membrane complex constitutes intermediate filament-like proteins (IMC proteins) that support the flattened alveolar sacs of the cortical cytoskeleton. These proteins are sequentially expressed and assembled into the cytoskeleton of the developing daughter cells (69, 70). Consistent with these previous findings, transcripts associated with these proteins in *S. neurona* exhibited varied levels across stages. The schizont stage transcriptome showed an enrichment of transcripts for SnIMC1 (SN3_01100800), SnIMC3 (SN3_00100240), SnIMC5 (SN3_00102755/6), SnIMC6 (SN3_00700100), SnIMC7 (SN3_02200165), SnIMC10 (SN3_02100415), and SnIMC15 (SN3_01700570) (Fig. 6B; Table S4). Three of these, SnIMC1, SnIMC6, and SnIMC10, were evident in the merozoite proteome (File S5). Highest transcript levels (TPM values and normalized read counts) for SnIMC15 (SN3_01700570) and SnIMC3 (SN3_00100240) were observed during the late schizont stage (72 h), consistent with their previously demonstrated presence during daughter cell budding in late endopolygeny (49, 71). Based on TPM values, the highest SnIMC13 transcript levels were observed in the merozoite stage.

Transcripts for homologs of all four IMC sub-compartment proteins (ISPs) identified so far in coccidia were present in the *S. neurona* transcriptome. Transcript levels of SnISP1 (SN3_00800805) did not vary between the merozoite and schizont development of *S. neurona*. On the contrary, SnISP2 (SN3_00600910), SnISP3 (SN3_01900535), and SnISP4 exhibited the highest transcript levels during the late schizont stage (Fig. 6B; Table S4), consistent with a role during daughter cell formation. Additionally, homologs of the glideosome-associated proteins (reviewed in reference 70), SnGAP40 (SN3_00400065), SnGAP45 (SN3_01700345), SnGAP50 (SN3_00300080), SnGAPM3 (SN3_03300135), SnGAPM1a, SnGAPM2B (SN3_00501370), and a putative SnGAPM, exhibited a significant increase in transcript levels during schizont development and more distinctly at the late schizont stage (Fig. 6B; Table S4), congruent with their role in daughter cell formation.

### AP2 transcription factors

Members of the phylum Apicomplexa express a family of conserved plant-like AP2 transcription factors (ApiAP2TFs) (72). In *T. gondii,* AP2TFs play an important role in parasite developmental biology by serving as activators and repressors of stage-specific gene regulation and cyst formation (19, 73, 74) and have been shown to be cell-cycle regulated (19). Analysis of transcript abundance across all time points of schizont development and the merozoite stage revealed 25 SnAP2TF genes that were significantly different in *S. neurona*. It was interesting to note that seven of the nine schizont-abundant AP2TFs belonged to AP2 group VII, while only one of the 18 merozoite-abundant AP2TFs belonged to AP2 group VII (Fig. 6C; Table S4). We also identified nine AP2TFs in *S. neurona* that are homologs of cell-cycle regulated TgAP2TFs (TgAP2X-5, TgAP2III-2, TgAP2XI-4, TgAP2XII-8, TgAP2XII-4, TgAP2VIII-4, TgAP2VIII-7, TgAP2VIIb-2, and TgAP2VIII-5). These TFs showed peak transcript levels during the S, C, or G1 phase of the *T. gondii* cell cycle. We did not detect any *S. neurona* homologs of TgAP2TFs from the M phase transcripts, which might be due to the schizont time-point series ending with late schizont, wherein the parasite has not yet initiated karyokinesis and cytokinesis to form the daughter cells.

Our two differential expression analysis methods disagreed regarding the transcript levels of *S. neurona* homologs of TgAP2VIIb-1, TgAP2IX-9, and TgAP2XII-5. In *T. gondii,* these are putative bradyzoite AP2 factors, and it would be difficult to speculate the role of bradyzoite AP2 factors in the merozoite and schizont stages of *S. neurona*.

Transcripts for SnAP2XI-5 and SnAP2X-5 were identified in the *S. neurona* transcriptome. *Toxoplasma* homologs, TgAP2XI-5 (75) and TgAP2X-5 (76), have been shown to work in coordination with each other to regulate the expression of virulence genes such as rhoptry and microneme proteins in *Toxoplasma* tachyzoite stage. Transcript levels of SnAP2XI-5 were similar in the merozoite and schizont stages. On the other hand, SnAP2X-5 showed significantly higher transcript levels in the late schizont and merozoite stages. Overall, the transcription patterns of AP2TFs in *S. neurona* were similar to that of *T. gondii* and might have comparable roles in the developmental biology and regulation of virulence factors of *S. neurona*. The *S. neurona* merozoite proteome showed evidence for proteins of AP2XII-4 (SN3_00501260), AP2IX-5 (SN3_00103370), and AP2X-9 (SN3_01000530) (Table S3).

### *Sarcocystis neurona* protein kinases

Apicomplexans possess a variety of protein kinases (PKs) that mediate infection and pathogenicity through signaling processes and are considered potential drug targets. In *T. gondii,* most PKs fall under three groups: cell cycle-associated protein kinases (CMGC), calcium/calmodulin-regulated kinases (CAMK), and rhoptry kinases (77, 78). A previous survey of the *S. neurona* genome revealed homologs to 97 apicomplexan PKs that fell under the abovementioned groups (79). Cell-cycle-associated protein kinases are involved in signaling and cell cycle control or regulation aspects. This group includes cyclin-dependent kinases (CDK), mitogen-activated protein kinases, and CDK-like kinases. The *S. neurona* transcriptome investigation revealed variation in transcript levels of these proteins in merozoite and schizont stages (Fig. 6D; Table S4). Given the complex intracellular development of *S. neurona*, it is unsurprising to find distinct CDK transcripts to be specifically abundant either in merozoite or schizont stages. Additionally, the gene for aurora kinase, involved in cell division by dispensing genetic material to daughter cells (80), was extensively transcribed in the late schizont stage, consistent with its predicted role in cell division. The second largest group of PKs identified in Apicomplexa are the calcium/calmodulin-regulated kinases that facilitate calcium signaling crucial in parasite invasion, egress, and other biological processes (81). Since mammals lack apparent homologs to CDPKs, these are also attractive targets for drug discovery (82). The use of bumped kinase inhibitors (BKIs) believed to target CDPK1 in *in vitro* assays led to marked defects in *S. neurona* invasion and growth, and *in vivo* assays in experimentally infected mice demonstrated efficacy of the BKI for reducing clinical signs (83). The *S. neurona* transcriptome revealed that several CDPK transcripts, viz., SnCDPK7 (SN3_02400475), SnCDPK8 (SN3_00900015), SnCDPK2 (SN3_00600795), SnCDPK2a (SN3_00501425), SnCDPK3 (SN3_00600020), SnCDPK6 (SN3_02900045), SnCDPK9 (SN3_00800640), and SnCDPK4a (SN3_01600345), were abundant in merozoites (Fig. 6D; Table S4). In contrast, transcript levels of a single putative CDPK (SN3_01400080) were substantially increased in the schizont transcriptome, with the highest levels of transcripts at the late schizont stage. At least two merozoite-abundant CDPKs (CDPK2a and CDPK3) were also present in the merozoite proteome (Table S3). Additionally, PKs belonging to other groups, such as the casein kinases, tyrosine-like kinases, and FIKK kinase, also showed stage-specific variation in transcript levels.

### Ubiquitin-proteasome system and autophagy

The ubiquitin-proteasome system (UPS) and autophagy are important for maintaining cellular homeostasis through protein and organelle degradation. Ubiquitin-conjugated single proteins, marked for proteolysis, are degraded in the barrel-shaped proteasomes. In contrast, autophagy occurs by forming an autophagosome that engulfs and degrades complex substrates such as protein aggregates and organelles (84). In parasitic protozoa, the components of the UPS are involved in biological processes such as morphological differentiation, replication, immune interaction, and virulence (85). The UPS is also suggested to be a potential and novel target for antiparasitic drug therapy (86). The *S. neurona* transcriptome showed a differential abundance of gene transcripts related to the UPS and autophagy in the merozoite and schizont stages. Interestingly, both TPM values and normalized read counts suggest that gene transcripts functioning in the process of ubiquitylation/deubiquitylation were significantly increased in the merozoite stage, while transcripts related to proteasomes were abundant in the schizont stage (Fig. 6E; Table S4). Altogether, transcripts of 38 different proteins involved in the UPS were found to be significantly abundant in the merozoite (Table S4) and included E2 ubiquitin-conjugating enzymes, E3 ubiquitin-protein ligases, ubiquitin transferases, ubiquitin-like proteins, ubiquitin fold-modifiers, ubiquitin thioesterase, ubiquitin-specific proteases, ubiquitin carboxyl-terminal hydrolase, and OTU family cysteine proteases. Gene transcripts across different time points of schizont development showed significantly increased TPM values and normalized read counts for genes encoding proteasome components such as the alpha and beta subunits, proteasome maturation factors, and proteasome-related proteases and hydrolases. Interestingly, all proteasome subunits identified in the schizont transcriptome show similar transcript levels throughout intracellular development (early-mid-late schizonts). In *T. gondii*, the autophagy-related proteins are involved in crucial non-canonical roles in intracellular development (87) and organelle inheritance (88) during daughter cell formation. Investigation of *S. neurona* autophagy-related proteins revealed variable transcript levels for three *T. gondii* homologs: SnATG9 (SN3_02000505), SnATG3 (SN3_00100680), and SnATG8 (SN3_00202075) and an additional *S. neurona* autophagy protein, apg6 (SN3_03400240), with no homologous sequence in *T. gondii*. Normalized read counts and TPM values of these SnATG gene transcripts are significantly fewer during the early and mid-schizont stages but tend to increase at the late-schizont stage and peak in the merozoite stage. The implications of this expression pattern remain to be determined. In *T. gondii*, ATG9 is involved in autophagy initiation (89), and ATG3 facilitates the conjugation of ATG8 to the autophagosome and is involved in mitochondrial maintenance (87, 88). It seems likely SnATGs play a crucial role in *S. neurona* endopolygeny.

### Differential transcript abundance across schizont development time points

**Fig 7:**
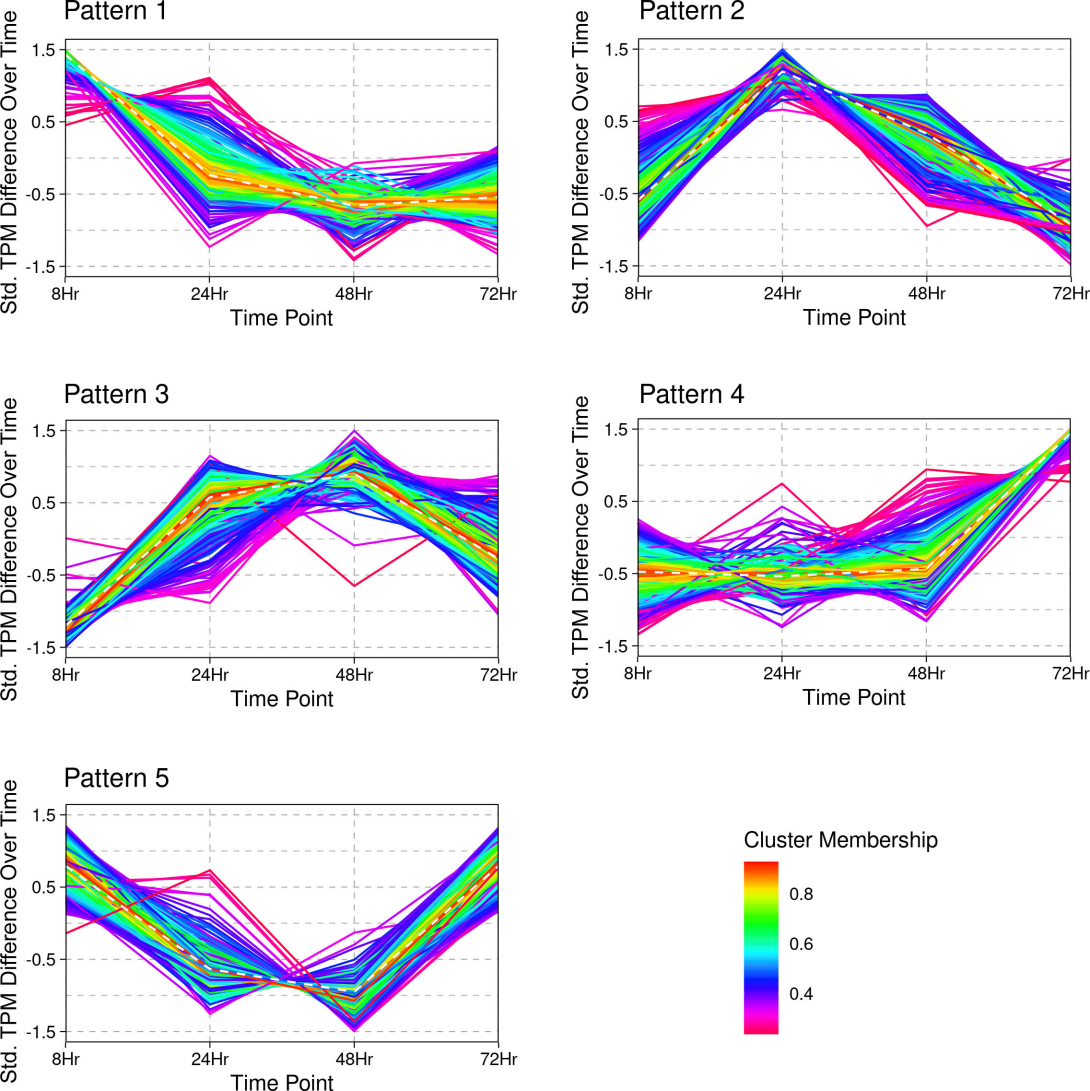
A representative example of gene transcript pattern clustering among differentially abundant schizont genes. TPM values were normalized so that the average value for each transcript level was zero and the standard deviation of its profile was one. Merozoite was used for comparison (0 h time point). Each line represents a transcript, and the color indicates each transcript’s membership value (between 0 and 1) and reflects the degree to which each member of the pattern either belongs or deviates from the cluster centroid.

In addition to comparing transcript abundance between the extracellular merozoite and the intracellular schizont, we also compared transcript levels across the four intracellular time points sampled (8, 24, 48, and 72 h), with the expectation that transcript abundance would change to meet developmental needs as *S. neurona* progressed through endopolygeny. Expression analysis was performed using TPM values and normalized read counts. Based on TPM values, transcript levels of 1,655 genes appeared to be variable during at least one time point during the intracellular development of *S. neurona* (Table S5). Using C-means clustering, these transcripts were assigned to five clusters with distinct expression patterns. The transcripts within each cluster showed peak levels at a particular time point during endopolygeny (Fig. 7; Table S5) and are described here.

Pattern 1 (cluster 2) had 428 genes, and their expression peaked at 8 h, followed by significantly lower TPM values during 24–48–72 h time points. Some of the known genes that followed this pattern included those involved in DNA processes, membrane transport molecules, rhoptry proteins, and kinases, viz., SnROP20 (SN3_00202375), SnROP21 (SN3_02000120), SnROP26 (SN3_00700495), and SnROP9 (SN3_00300585). Three microneme genes, two annotated as SnMIC8 (SN3_02200145 and SN3_00400805) and one as SnMIC13 (SN3_00800900), three SnAP2TFs (AP2ix-5, AP2ix-9, and AP2viii-7), along with subtilisin1 (SN3_00101610), several CDPKs, and serpins were among the known genes in this cluster. Based on the expression pattern, it can be speculated that a significant increase in the transcript levels of genes in these clusters may have begun in the mature schizont stage and must be of functional importance in the merozoite, the merozoite-schizont transition, or early schizont development.

In pattern 2 (cluster 5), the transcript levels were highest during the early schizont stage at 24 h and showed a gradual decline in abundance through the mid- and late-schizont stages (48 and 72 h). Gene transcript levels peaking at the 24-h time point suggest that these gene products may be required for establishing the niche for schizont development beyond the transition from a merozoite to a schizont stage. This cluster included 368 genes, and some examples include genes encoding proteins, such as DNA-directed RNA polymerase, RNA exonuclease, mRNA turnover-related proteins, tRNA and rRNA proteins, translation initiation and elongation-related proteins, and mitochondria-associated proteins. Surprisingly, transcripts for a SnMIC8 paralog (SN3_00401485) and a rhoptry protein annotated as SnROP21 (00800370) showed the highest level at 24 h into schizont development. The cytoskeletal protein SnIMC7 belonged to this cluster, and the abundance of SnIMC7 (SN3_02200165) transcripts during early-mid schizont stages (24 and 48 h) during *S. neurona* endopolygeny contradicts the previous speculation that its expression pattern might have diverged from TgIMC (71). Nonetheless, the *in silico* analyses and cluster grouping are subject to change depending on the parameters set for gene clustering.

There were 282 genes whose transcript levels gradually increased during 8–24–48 h time points. At the late-schizont stage (72 h), there was a slight decrease in TPM values but remained higher than at the 8-h time point (pattern 3 or cluster 4). The pattern suggests that these gene products might be required at increasing levels as the mother cell enlarges throughout the process of intracellular development. This cluster’s known genes include certain DNA polymerases and replication factors, histones, proteasome proteins, and AP2 factors (SnAP2ix-9, SnAP2viii-5, and SnAP2x-9). Also included in this cluster were transcripts encoding the “aaa-family proteins” that play a role in various cellular processes (90), such as DNA replication, protein metabolism, organelle biosynthesis, and intracellular transport consistent with endopolygeny.

Patterns 4 and 5 (clusters 1 and 3) were similar in that transcript levels peaked in the late-schizont (72-h time point), but their levels in the early schizonts differed between the two patterns (Fig. 7). The 341 genes of pattern 4 showed notably higher transcript levels at 72 h compared to the 8-h time point of schizont development. On the contrary, the cluster of 236 genes in pattern 5 showed similar transcript levels at both 8 and 72 h. Based on this difference, genes with pattern four expression could be involved in the final steps of endopolygeny, especially the formation and assembly of the nascent merozoites and their egress from and entry into the host cell. In contrast, the genes in pattern 5 (cluster 3) might be required to form the nascent daughter merozoites, their egress from the host cells, and/or entry into the new host cell. Some of the homologs of well-characterized genes of *T. gondii* were found in these two clusters. Examples of genes clustering in pattern 4 include those of several myosins (MyoH, MyoJ, MLC1, and MLC2), alpha- and beta-tubulin, dyenins, perforin-like proteins, several IMCs (IMC1, IMC3, IMC6, IMC10, IMC14, and IMC15), IMC sub-compartment proteins (isp1, isp2, and isp3), glideosome-associated proteins, protein kinase CMGC, AP2 transcription factors (ap2iii-2, ap2xii-2, ap2ix-5, ap2x-5, and ap2xii-9), and SnGRA9. Genes clustering in pattern 5 include myosins [myoA (SN3_00301710, SN3_01800105) and myoC (SN3_03400245)], subtilisin SUB2, AP2TFs (SnAP2Xi-4 and AP2xii-5), microneme proteins (MIC1, MIC10, and MIC12), formin FRM3, rhomboid protein ROM1, TLN4, and protein kinase CAMK. Transcripts for ROP35, a rhoptry kinase of unknown function, were abundant at the late-schizont stage.

Overall, cluster analysis of the intracellular development stages showed that the schizont undergoes active gene expression changes across endopolygeny, and the gene sets being transcribed were distinct from the merozoite stage. While we generated only five clusters, these data on https://Sarcocystis.shinyapps.io/SN3_Gene_Expression_Clustering/ allow researchers to adjust the number of clusters and other parameters as needed and provides for analysis at a greater depth.

### *Sarcocystis neurona*-unique hypothetical proteins

Both SN3.E1 and SOSN1 (18) genomes contain a significant proportion (~20%) of annotated genes with no detectable homolog in the public databases. Also, analysis of the RNA-Seq data revealed additional transcripts from the non-annotated regions of the genome. BLASTX searches of these merozoite and schizont transcripts against NCBI’s non-redundant (nr) protein database revealed no similarity to any of the deposited sequences, suggesting that these are unique to *S. neurona* or the genus *Sarcocystis*. Of the 1,223 predicted transcripts with no match in NCBI’s nr protein database, 11 were predicted to contain no open reading frame and may represent putative non-coding genes. A total of 826 and 397 of these unique gene transcripts were abundant in the merozoite and schizont transcriptomes, respectively (Table 1). Of these 826 merozoite-abundant transcripts, only 23 were evident in the merozoite proteome data (Table S3). Functional annotation on the Blast2GO platform suggested no conserved domain or motif hit to several of these *S. neurona* unique genes. However, putative signal peptides and sequences with transmembrane and/or cytoplasmic regions were predicted for a small portion of the transcripts (Table 1; Table S6). Transcripts of these *S. neurona* unique genes were distributed across all developmental stages and/or time points examined in this study.

**TABLE 1:** Blast2GO-based InterPro domain analysis of putative *Sarcocystis* unique genes

	Total	No IPS match	Signal P	Interpro GO IDs	Transmembrane	Cytoplasmic/noncytoplasmic
Schizont						
Annotated	224	26	66	16	55	97
Unannotated	173	85	30	2	35	48
Merozoite						
Annotated	308	49	61	8	67	114
Unannotated	518	279	68	3	73	127

Given their apparent restriction to *Sarcocystis*, these uncharacterized genes might play a role in the unique biology of this parasite. For example, *S. neurona* resides free in the host cell’s cytoplasm and is therefore exposed to the innate host defenses in the cytosol. This is unlike *T. gondii*, which is surrounded by a PV that compartmentalizes the parasite and protects it against such insults. The *T. gondii* PV likely serves various functions (15, 91), including nutrient transport to the parasite. In contrast, *S. neurona* has ready access to these resources and likely requires a different repertoire of proteins to assist in nutrient acquisition. In this context, it is also interesting to note that only a few homologs of *T. gondii* GRA proteins were evident in the *S. neurona* transcriptomes and genome annotations (18). It seems conceivable that the dense granule contents of *S. neurona* are different from *T. gondii* or *N. caninum* and might include proteins encoded by a subset of these currently unknown sequences. The temporal expression and preliminary protein predictions outlined herein provide the initial bases for the investigation of these factors that are seemingly unique to *Sarcocystis*.

### Conclusions and future directions

*Sarcocystis* is an extensive genus comprising more than 200 species that infect a wide variety of animals and birds. The biology, morphology, host range, mode of replication, etc., are unique to this genus, but we only have limited knowledge of the molecular mechanisms responsible for the lifestyle of organisms classified in this genus. Within this genus, the equine pathogen *Sarcocystis neurona* is a well-studied member, and the genome sequence for this species (18) identified conserved genes, SPDs involved in attachment and invasion, unique metabolic enzymes, etc., but more importantly, it revealed the novelty of genes in this parasite. The transcriptome analyses presented here provide evidence for differential transcript abundance in merozoite and schizont stages and confirm the active transcription of *Sarcocystis*-unique genes. A relatively unexplored area in the host-pathogen interaction of *S. neurona* is its interaction with and regulation of the host. Proteome, genome, and transcriptome data have clearly demonstrated the expression of ROP proteins in a rhoptry-less parasite stage and the divergence of dense granule proteins. In other apicomplexans, both protein groups contribute to establishing the niche for parasite survival and interaction with the host. Studying the role of these proteins in *S. neurona* will enhance our understanding of intracellular development. Additionally, the proteomic data generated from the merozoite stage corroborates some of the transcriptomic evidence and prompts the need for further experimental validation to understand their role in the biology of *S. neurona*. In addition to the basic molecular tools that are available for the genetic manipulation of this parasite, we now have the capability to perform gene disruptions using CRISPR/Cas9 (92) and select mutants using an HXGPRT-based positive-negative selection system (93). We recently established the feasibility of CRISPR/Cas9-guided epitope tagging in *S. neurona* (S. Dangoudoubiyam and D. K. Howe, unpublished data), thus expanding this parasite’s molecular genetics tool kit and paving the way to initiate functional studies. We expect that the “-Omics” data and the molecular tools available for *S. neurona* will enable experimentation of potentially interesting areas in the biology of this parasite and the genus *Sarcocystis*.

## SUPPLEMENTAL MATERIAL

Supplemental table captions. msphere.00111-24-s0001.docx Captions.

Table S1 msphere.00111-24-s0002.xlsx RNA-Seq read mapping and alignment.

Table S2 msphere.00111-24-s0003.xlsx Merozoite and schizont abundant transcripts.

Table S3 msphere.00111-24-s0004.xlsx Merozoite transcripts and proteome evidence.

Table S4 msphere.00111-24-s0005.xlsx *S. neurona* gene families.

Table S5 msphere.00111-24-s0006.xlsx Schizont gene clustering.

Table S6 msphere.00111-24-s0007.xlsx Gene ontology.

ASM does not own the copyrights to Supplemental Material that may be linked to, or accessed through, an article. The authors have granted ASM a non-exclusive, world-wide license to publish the Supplemental Material files. Please contact the corresponding author directly for reuse.
