# Supplementary material for: Temporal gene expression during asexual development of the apicomplexan Sarcocystis neurona
Source: mSphere. 2024 May 29;9(6):e00111-24. doi: 10.1128/msphere.00111-24 (PMC11332336; doi:10.1128/msphere.00111-24)
Supplement: Supplemental table captions. — Captions. [file msphere.00111-24-s0001.docx]

Table S1: Summary of read mapping and read alignment for RNA-seq generated from merozoite and schizont development stages of *S. neurona.*

Table S2: List of merozoite and schizont genes with abundant transcript levels resulting from comparison of TPM values of merozoite stage against schizont time points (individual or a combination of time points).

Table S3: List of *S. neurona* merozoite abundant transcripts with evidence of protein expression.

Table S4: List of transcripts belonging to gene families important to the Apicomplexa.

Table S5: List of clustered schizont genes and their transcript levels at different time points.

Table S6: List of *S. neurona* genes with no BLAST hit.
